# Investigating patellar motion using weight-bearing dynamic CT: normative values and morphological considerations for healthy volunteers

**DOI:** 10.1186/s41747-024-00505-6

**Published:** 2024-09-19

**Authors:** Luca Buzzatti, Benyameen Keelson, Savanah Héréus, Jona Van den Broeck, Thierry Scheerlinck, Gert Van Gompel, Jef Vandemeulebroucke, Johan De Mey, Nico Buls, Erik Cattrysse

**Affiliations:** 1 School of Allied Health, Anglia Ruskin University (ARU), Cambridge, UK; 2 Experimental Anatomy Research Group (EXAN), Vrije Universiteit Brussel (VUB), Brussels, Belgium; 3 Department of Radiology, Vrije Universiteit Brussel (VUB), Universitair Ziekenhuis Brussel (UZ Brussel), Brussels, Belgium; 4 Department of Electronics and Informatics (ETRO), Vrije Universiteit Brussel (VUB), Brussel, Belgium; 5 imec, Leuven, Belgium; 6 Department of Orthopaedic Surgery and Traumatology, Vrije Universiteit Brussel (VUB), Universitair Ziekenhuis Brussel (UZ Brussel), Brussels, Belgium

**Keywords:** Healthy volunteers, Joint instability, Patella, Tomography (x-ray computed), Weight-bearing

## Abstract

**Background:**

Patellar instability is a well-known pathology in which kinematics can be investigated using metrics such as tibial tuberosity tracheal groove (TTTG), the bisect offset (BO), and the lateral patellar tilt (LPT). We used dynamic computed tomography (CT) to investigate the patellar motion of healthy subjects in weight-bearing conditions to provide normative values for TTTG, BO, and LPT, as well as to define whether BO and LPT are affected by the morphology of the trochlear groove.

**Methods:**

Dynamic scanning was used to acquire images during weight-bearing in 21 adult healthy volunteers. TTTG, BO, and LPT metrics were computed between 0° and 30° of knee flexion. Sulcus angle, sulcus depth, and lateral trochlear inclination were calculated and used with the TTTG for simple linear regression models.

**Results:**

All metrics gradually decreased during eccentric movement (TTTG, -6.9 mm; BO, -12.6%; LPT, -4.3°). No significant differences were observed between eccentric and concentric phases at any flexion angle for all metrics. Linear regression between kinematic metrics towards full extension showed a moderate fit between BO and TTTG (*R*^2^ 0.60, β 1.75) and BO and LPT (*R*^2^ 0.59, β 1.49), and a low fit between TTTG and LPT (*R*^2^ 0.38, β 0.53). A high impact of the TTTG distance over BO was shown in male participants (*R*^2^ 0.71, β 1.89) and patella alta individuals (*R*^2^ 0.55, β 1.91).

**Conclusion:**

We provided preliminary normative values of three common metrics during weight-bearing dynamic CT and showed the substantial impact of lateralisation of the patella tendon over patella displacement.

**Relevance statement:**

These normative values can be used by clinicians when evaluating knee patients using TTTG, BO, and LPT metrics. The lateralisation of the patellar tendon in subjects with patella alta or in males significantly impacts the lateral displacement of the patella.

**Key Points:**

- Trochlear groove morphology had no substantial impact on motion prediction.
- The lateralisation of the patellar tendon seems a strong predictor of lateral displacement of the patella in male participants.
- Participants with patella alta displayed a strong fit between the patellar lateral displacement and tilt.
- TTTG, BO, and LPT decreased during concentric movement.
- Concentric and eccentric phases did not show differences for all metrics.

**Graphical Abstract:**

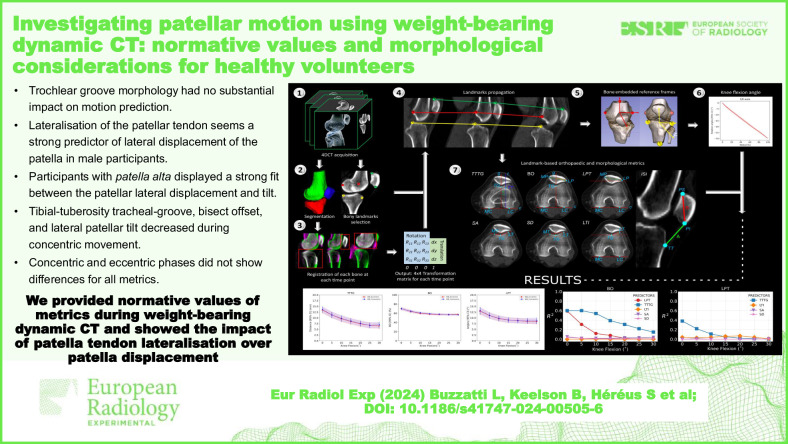

## Background

Patellar instability refers to a subluxation or abnormal patella tracking in the trochlear groove. The prevalence is between 5 and 77 per 100,000 people, and adolescents between 14 years to 18 years are mostly affected [1–4]. Patella dislocations represent 2% to 3% of traumatic knee injuries with a high risk (15–45%) of further recurrence after the first episode [5].

To evaluate patellar instability, we can measure morphology-based metrics such as: the axial distance between the tibial tuberosity tracheal groove (TTTG); the bisect offset (BO) (the proportion of the patella lateral to the midline of the femur); and the lateral patellar tilt (LPT) (the angle between the patella and the most posterior part of the femoral condyle) [6, 7]. The TTTG gives an indication of the lateralisation of the patellar tendon insertion on the tibial tuberosity relative to the deepest part of the trochlear groove, while BO and LPT estimate the position and inclination (tilt) of the patella relative to the femur. These morphological measurements are used during surgical planning and as postsurgical outcome measures.

Literature suggests that a TTTG between 15.5 mm and 20 mm, or above, is considered pathological [8] and may be an indication for surgical correction (*i.e*., distal realignment procedures). A patellar tilt angle ≥ 15° and a BO ≥ 57% are reported to be associated with full-thickness cartilage damage and pain [9]. However, most measurements are performed on non-weight-bearing and static images and these two factors can be considerable major limitations for different reasons. Firstly, patellofemoral kinematics observed in a supine position do not precisely reflect the joint motion experienced during weight-bearing activities [10]. Moreover, weight-bearing and non-weight-bearing acquisitions demonstrated substantial differences in hip–knee–ankle angle and line convergence angle [11], TTTG offset [12], knee joint alignment [13, 14], femoral external rotation [15], lateral patellar displacement [16], and patellofemoral joint contact [17]. Additionally, muscle activation is significantly affected when transitioning from a weight-bearing to a non-weight-bearing position [18]. Secondly, static acquisitions may not fully capture the bone-to-bone interaction and muscle activation during movements, leading to significant differences in joint kinematics [19].

Dynamic acquisitions can better portray patellar motion [20], can reproduce altered kinematics of unstable joints more realistically and can reduce false negatives [21, 22]. Metrics like the sulcus angle (SA), sulcus depth (SulDe), medial and lateral trochlear inclination (LTI), congruence angle, trochlear angle and presence of patella alta have been investigated in static or non-weight-bearing dynamic images in patients with patellofemoral disorders [23–26], but not in dynamic weight-bearing conditions.

Dynamic computed tomography (also known as four-dimensional CT) allows dynamic acquisition of moving joints with high temporal and spatial resolution. It takes advantage of the wide field of view (up to 16 cm) of modern wide-beam CT scanners and fast tube rotation speeds to visualise joints during movement. Several studies have investigated patella tracking during knee movements among individuals with specific conditions [27–29], or asymptomatic [28, 30] and healthy participants [31] using dynamic CT. However, as emphasised in a recent review [32], all investigations were conducted in a non-weight-bearing position.

To understand pathological patterns or the impact of surgical procedures in restoring normal kinematics of the knee joints, it is necessary to first understand how healthy individuals move. Therefore, this study aimed to investigate the patellar motion of healthy participants in weight-bearing conditions via dynamic CT scanning and provide preliminary normative values for TTTG, BO, and LPT. These values will serve as a potential reference for comparing healthy to pathological conditions. The study’s second aim was to explore the relationship and impact of morphological characteristics of the trochlear groove on patellar tracking.

## Methods

### Participants

Healthy adult volunteers were recruited between June 2020 and February 2021. Participants reported no symptoms during activities of daily living at the level of the lower extremities in the last 6 months. Participants were excluded from the study if they had a history of lower limb surgery or any known orthopaedic or musculoskeletal conditions (*e.g*., anterior cruciate ligament or meniscal injuries, patellofemoral pain syndrome, instability, and osteoarthritis), if they were pregnant, or had any other contraindications for CT scanning. Each volunteer underwent an assessment (clinical examination, joint range of motion and main muscle group strength) by an experienced physiotherapist. If any abnormalities were identified during the clinical examination the participant was excluded from the study.

The study was approved by the local ethics committee (B.U.N 143201733617). Written informed consent was obtained from all participants included in the study.

### Setup

**Fig. 1:**
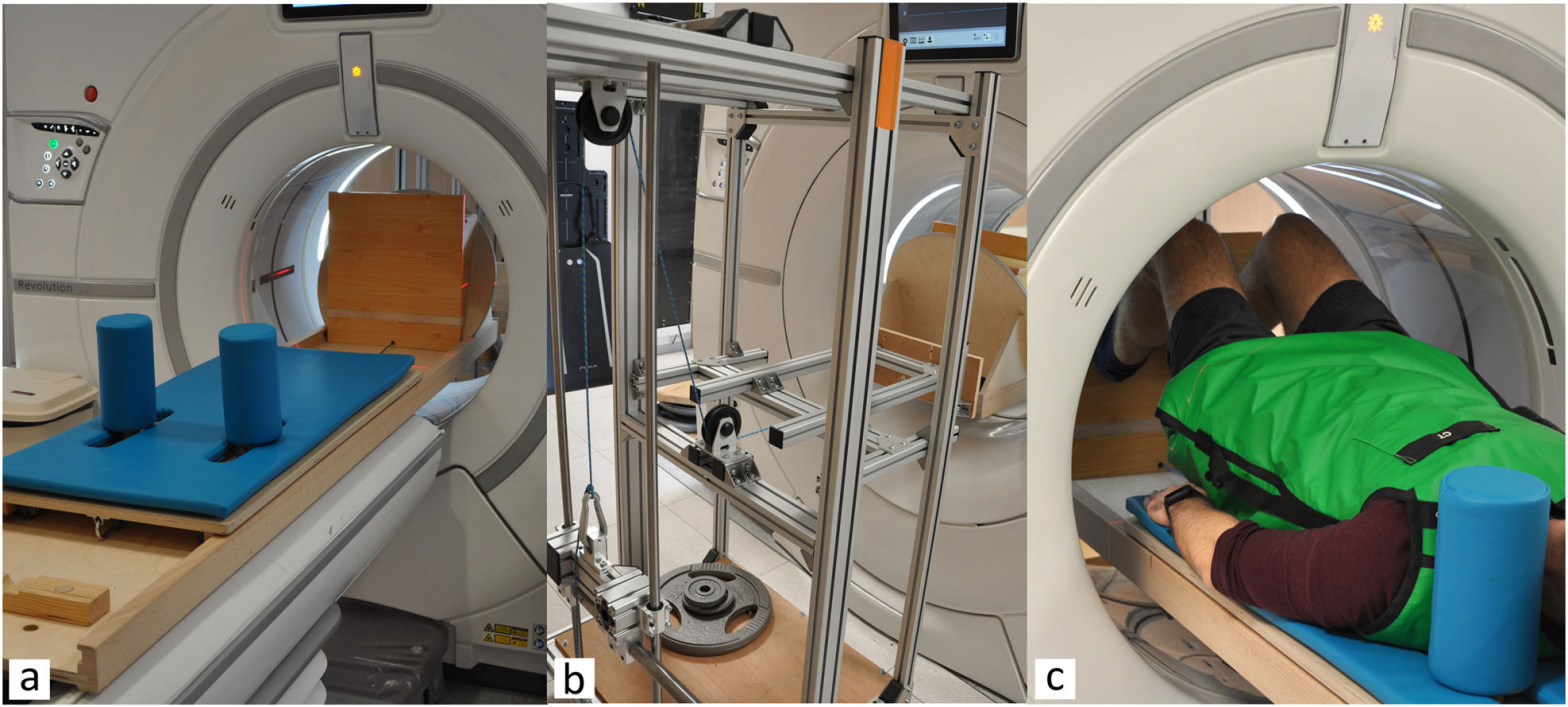
**a** Weight-bearing device; **b** counter-weight system; and (**c**) knee in flexion during the movement performed inside the gantry

A novel weight-bearing device was used to simulate constant gravitational force during horizontal dynamic CT acquisition. This device consisted of a fixed base that was attached to the bed of the CT; a sliding bed on which the subjects lay; a platform the subject used to position the feet and push against; and a counteracting system of weights and pulleys that ensured constant load during concentric and eccentric movements (Fig. 1a, b). The device was previously validated and consistently replicated an orthostatic (standing) squat when a counterweight equivalent to 42% of the participant’s body weight was used [33]. The weights used on the device (Fig. 1b) matched the target load with a precision of 0.25 kg.

Metrics such as TTTG can be influenced by the relative position between the femur and tibia (trochlear groove medialisation, tibial tuberosity lateralisation, or increased rotation) [34, 35]. In order to standardise the feet and leg position between participants, one of the researchers ensured that the participants positioned their feet straight with the second toe on top of the platform at an equal distance from the midline. The pelvis was positioned at the bottom portion of the sliding bed. This setup allowed for the standardisation of the participant’s position without affecting their normal knee motion. After wearing appropriate lead shielding, the participant was asked to perform a smooth and continuous movement of flexion–extension of the knees (Fig. 1c). Maximum flexion was determined either when participants felt their heels lifting from the platform or the knee touched the CT gantry. The participant was instructed to follow a metronome with a rhythm of 15 full cycles (from full extension to maximal flexion, back to full extension)/min. This rhythm ensured the capture of at least one full cycle during the 6.7-s scanning time.

### CT protocol and image processing

Dynamic images were acquired in continuous cardiac scanning mode on a wide beam CT (256-slice Revolution CT, GE Healthcare, Chicago, IL, USA) with a 50 cm field of view, tube voltage of 80 kVp and a tube current of 50 mA. Tube rotation time was 0.28 s with a slice thickness of 2.5 mm. Cardiac scanning mode (three cycles 0–300%, using a 30-bpm electrocardiographic simulation) has been shown to produce fewer motion artefacts than cine mode [36]. The participant performed flexion–extension movements in a squat-like motion with both knees in the scanner’s field of view. The effective dose for the whole dynamic acquisition was 0.02 mSv (CT dose index volume = 6.73 mGy) for all participants.

**Fig. 2:**
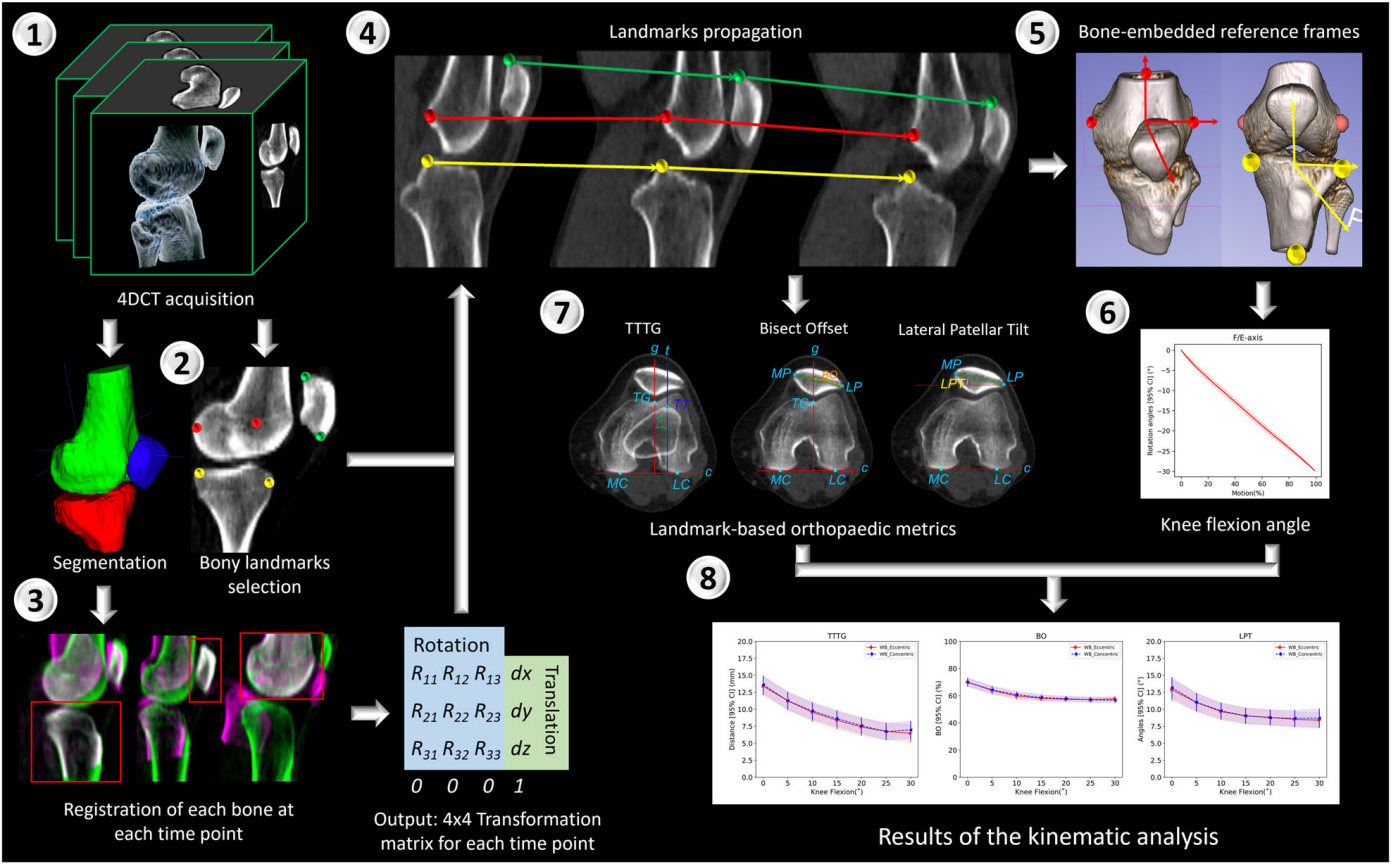
Data processing workflow. 1. Acquisition of four-dimensional CT images. 2. Automatic segmentation of the bones of interest and selection of bony landmarks. 3. Registration of each bone for every moving image to the fixed image (knee in fully extended position), which outputs a series of transformation matrices representing the amount of translation and rotation to align the two images. 4. Propagation of all bony landmarks for each time point. 5. Definition of bone-embedded reference frames based on the bony landmarks. 6. Calculation of the flexion angle of the knee from 0° to 30°. 7. Calculation of landmark-based orthopaedic kinematic metrics (TTTG, BO, and LPT) and morphology metrics (SA, SulDe, and LPI). 8. The result of each metric at different flexion angles

The data processing workflow, from the acquired images to the final results, is presented in Fig. 2.

To register the CT scan images, we generated a segmentation of the reference image (knee in full extension) to create a mask. This mask was then used to guide the pairwise rigid registration between the reference image and subsequent time points [37]. In order to optimise and reduce processing time, an automated multi-atlas multi-label segmentation approach was used to create the segmentations of the femur, tibia and patella. This approach has proven to be accurate and reliable for both automated segmentations and kinematic calculations [38]. The output of the automated workflow consisted of a series of transformation matrixes describing the change in the position of the bones of interest compared to the reference image. Segmentation and registrations were achieved using in-house code developed with Elastix [39] and the Insight Registration and Segmentation Toolkit (ITK 5.2.0, Kitware Inc., New York, USA).

### Morphological and kinematic metrics and analysis

Sixteen bony landmarks were selected by one researcher on the fixed reference image using the VV image viewer [40]. These landmarks were pinpointed as the outermost regions of the bone. We identified these landmarks accurately by verifying their positions in the three planes (sagittal, frontal, and horizontal). This procedure was aided by having the leg aligned with the *z*-axis of the CT machine. The manual selection of bony landmarks defining the femur and tibia coordinate system has shown low inter- and intra-observer variability [41] and minimal impact on kinematic calculations [38].

The spatial transformation obtained from the image registration step was used to propagate the selected landmarks to subsequent frames. This allows tracking the spatial change of such landmarks in time during knee motion. Moreover, because kinematic metrics were constructed based on bony landmarks it was possible to compute each metric at each time point.

**Fig. 3:**
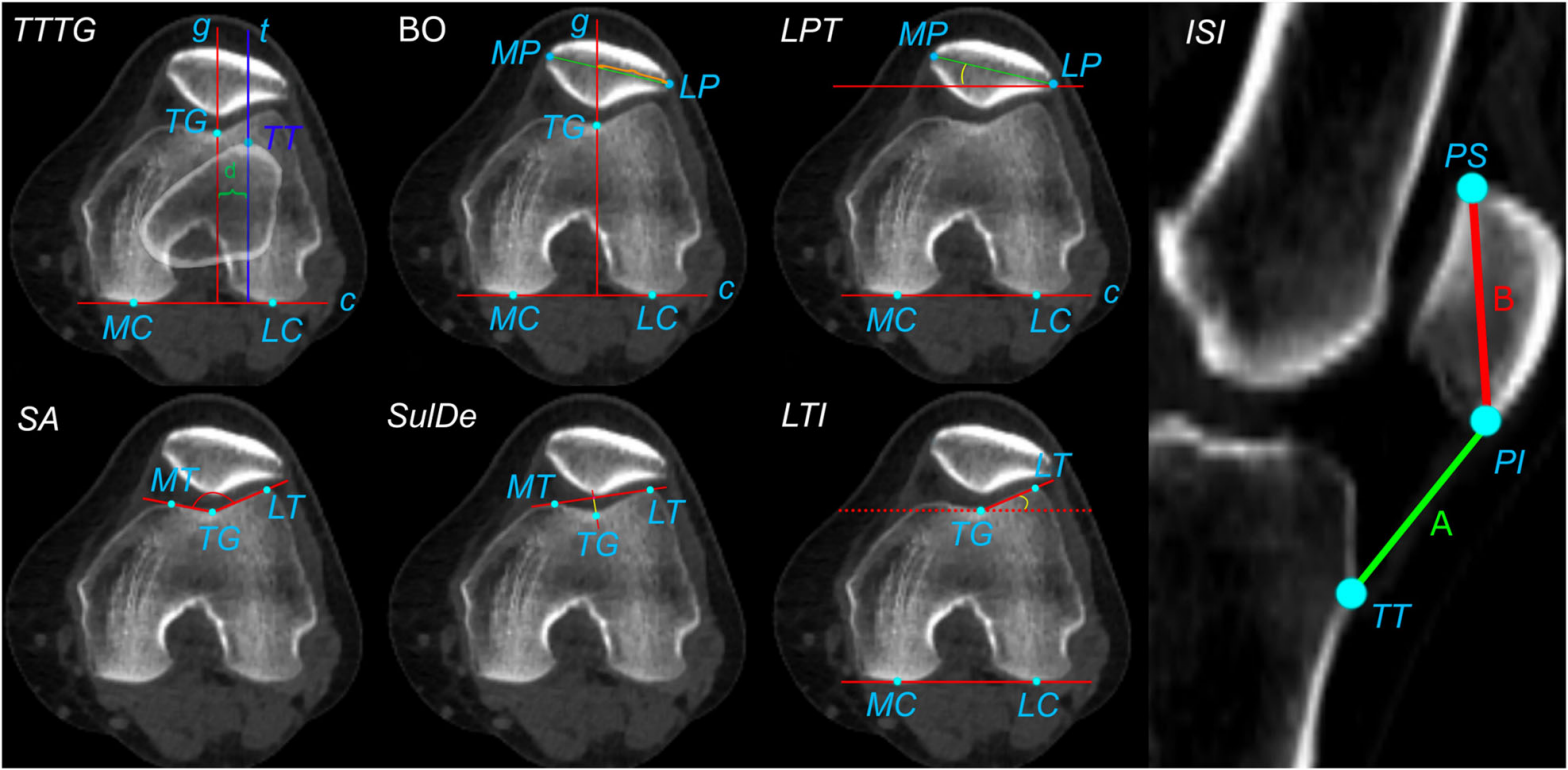
Bony landmarks: most posterior portion of the medial condyle (MC) and the lateral condyle (LC); deepest point of the trochlear groove (TG), the most anterior lateral (LT) and medial (MT) portions of the trochlear groove; the most medial (MP), lateral (LP), superior (PS), and inferior (PI) portion of the patella; the tibial tuberosity (TT). Kinematic metrics: the TTTG distance was defined as the distance (*d*) between the axis *g* and *t*. *g* is the axis passing through TG perpendicular to the axis passing through MC and LC (axis *c*). The *t* is the axis passing through TT and perpendicular to the *c*-axis. The BO was defined as the distance between LP and the intersection between *g* and the axis passing through MP and LP. This distance is expressed as a percentage of the mediolateral size of the patella (distance between MP and LP). The LPT was defined as the angle (°) between *c* and the axis passing through MP and LP. Morphological metrics: the SA was calculated as the angle (°) formed by the two axes passing through TG-LT and TG-MT, respectively. SulDe was calculated as the perpendicular distance (mm) between TG and the axis passing through LT and MT. LTI was calculated as the angle (°) between the axis *c* (MC-LC) and the axis passing through TG-LT. The ISI was calculated as the ratio A/B, where A is the patellar-tendon length (TT-PI) and B is the patellar length (PI-SI)

We choose three kinematic metrics (Fig. 3): the TTTG distance [42], the BO [43], and the LPT [44]. These are commonly used in radiology and orthopaedics to portray knee and patella kinematics during surgical planning. SA, SulDe, and LTI were selected as metrics that describe the trochlear groove morphology. Finally, the Insall–Salvati index (ISI) was calculated to identify participants with patella alta.

For each of the dynamic datasets, the knee flexion, abduction-adduction and rotation angles were calculated based on the tibia and femur bone-embedded reference frames. The rotation of the knees was comparable across the individuals and showed a relatively small confidence interval (CI) for the tibiofemoral rotations, with a margin of error of around ± 1°.

Each of the three kinematic metrics was described as a function of the flexion angle of the knee. The ranges of the first 30° of flexion and the last 30° of extension hold significant importance in studying conditions like patellar instability. These specific angles offer key insights into potential pathologies and are particularly valuable for comprehensive analysis [18]. Therefore, 30° of flexion was chosen as a cutoff for the analysis. Two phases were analysed and compared: the eccentric phase (from full extension to knee flexion) and the concentric phase (from knee flexion to full extension).

### Statistical analysis

Descriptive statistics (mean and 95% CI) for both the eccentric and the concentric phases were reported from 0° to 30° of knee flexion at each 5-degree increment. Visual inspection of the 95% CI was used to identify differences between eccentric and concentric phases.

Linear regression was computed to explore the relationship, predictive strength and impact between variables. BO and LPT were used as dependent variables as they represent the motion of the patella. Since the different morphology metrics (SA, SulDe, and LTI) used the same landmarks for their calculations, the correlation between them was very high. Therefore, it was not indicated to perform a linear regression between them. They were used as predictor variables only. Group stratification based on sex and the presence of patella alta was also computed, as the latter showed to have a potential impact on patella tracking. The patella alta cut-off based on the ISI was set at 1.3 [45]. The results of the regression model were expressed as the coefficient of determination (*R*^2^), statistical significance of the model (α = 0.05), residual standard deviation, β coefficient and standard error of the β coefficient (βSE). Results were interpreted based on correlation coefficient cutoffs [46].

## Results

**Fig. 4:**
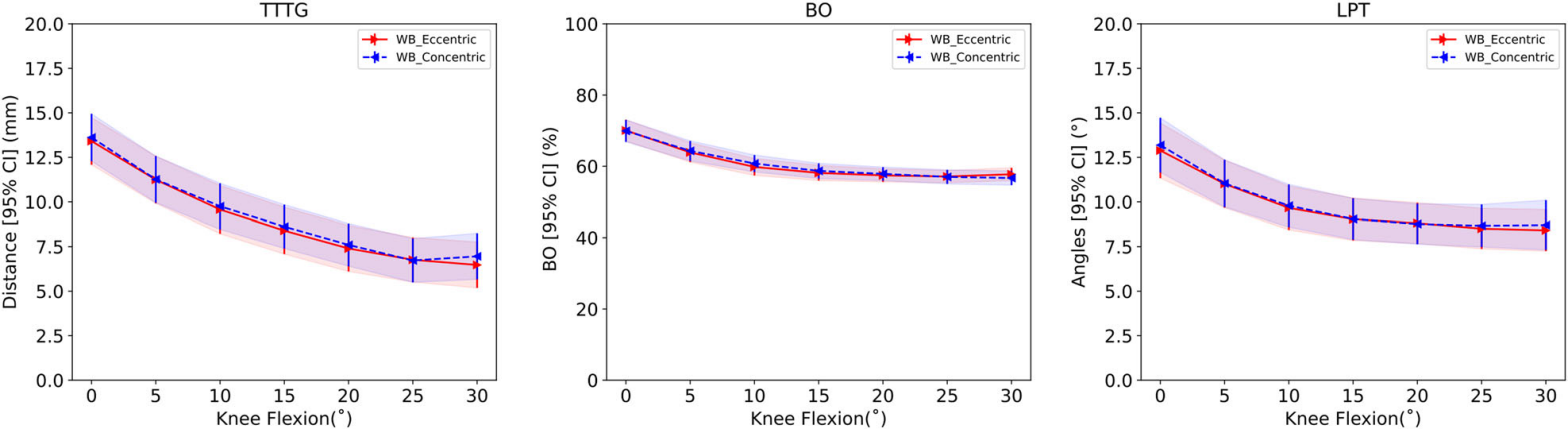
The TTTG, BO, and LPT from 0° to 30° of knee flexion. The red line represents the eccentric phase while the blue lines represent the concentric phase, during weight bearing. The shaded area represents the 95% CI of each curve

Forty-two knees from 21 subjects (nine females and twelve males) were included in the analysis (Table 1). In the first 30° of flexion, all the calculated metrics (TTTG, BO, and LPT) showed a decreasing trend during the eccentric phase (knee flexion) and an increasing trend during the concentric phase (knee extension) (Table 2 and Fig. 4).

**Table 1:** Participant anthropometric characteristics and morphological characteristics at 0° flexion

	Overall, (*n* = 21)	Females, (*n* = 9)	Males, (*n* = 12)
Age (years)	29.0 (11.0)	26.0 (8)	32.0 (12)
Weight (kg)	75.0 (13.5)	64.0 (7)	83.0 (11.3)
Height (cm)	176.3 (7.8)	170.0 (5.9)	181.0 (4.8)
Body mass index	24.0 (3.4)	22.2 (2.3)	25.5 (3.4)
LTI (°)	21.0 (6.5)	18.2 (6.3)	22.1 (6.9)
SulDe (mm)	5.9 (1.5)	5.4 (1.8)	6.1 (1.3)
SA (°)	138.4 (7.6)	140.7 (7.9)	137.4 (7)
ISI	1.36 (0.20)	1.27 (0.13)	1.30 (0.21)

Data are given as mean (standard deviation)

**Table 2:** Mean TTTG, BO, and PT and corresponding 95% CIs at different knee flexion angles for the concentric and eccentric phase of the motion

Flexion, (°)	TTTG, (mm)		BO, (%)		LPT, (°)	
Movement	Eccentric	Concentric	Eccentric	Concentric	Eccentric	Concentric
0°	13.4 [12.1–14.7]	13.6 [12.3–14.9]	70.1 [67.0–73.1]	69.9 [66.8–73.0]	12.9 [11.3–14.4]	13.2 [11.7–14.7]
5°	11.2 [9.9–12.6]	11.3 [10.0–12.6]	63.9 [61.2–66.6]	64.3 [61.5–67.1]	11.0 [9.7–12.3]	11.0 [9.7–12.4]
10°	9.6 [8.2–10.9]	9.8 [8.5–11.0]	59.8 [57.4–62.2]	60.8 [58.4–63.1]	9.7 [8.4–10.9]	9.8 [8.6–11.0]
15°	8.4 [7.1–9.7]	8.6 [7.4–9.8]	58.1 [56.0–60.3]	58.7 [56.6–60.8]	9.0 [7.8–10.2]	9.0 [7.9–10.2]
20°	7.4 [6.1–8.7]	7.6 [6.4–8.8]	57.4 [55.6–59.3]	57.9 [56.0–59.7]	8.8 [7.7–10.0]	8.8 [7.6–9.9]
25°	6.8 [5.5–8.0]	6.7 [5.5–8.0]	57.1 [55.4–58.8]	57.0 [55.0–59.0]	8.5 [7.4–9.6]	8.7 [7.5–9.9]
30°	6.5 [5.2–7.8]	7.0 [5.7–8.2]	57.7 [55.9–59.6]	56.7 [54.7–58.7]	8.4 [7.3–9.6]	8.7 [7.3–10.1]

*TTTG* Tibial tuberosity-tracheal groove, *BO* Bisect offset, *LPT* Lateral patellar tilt, *Flexion* kknee flexion angle expressed in degrees from 0° to 30°

**Fig. 5:**
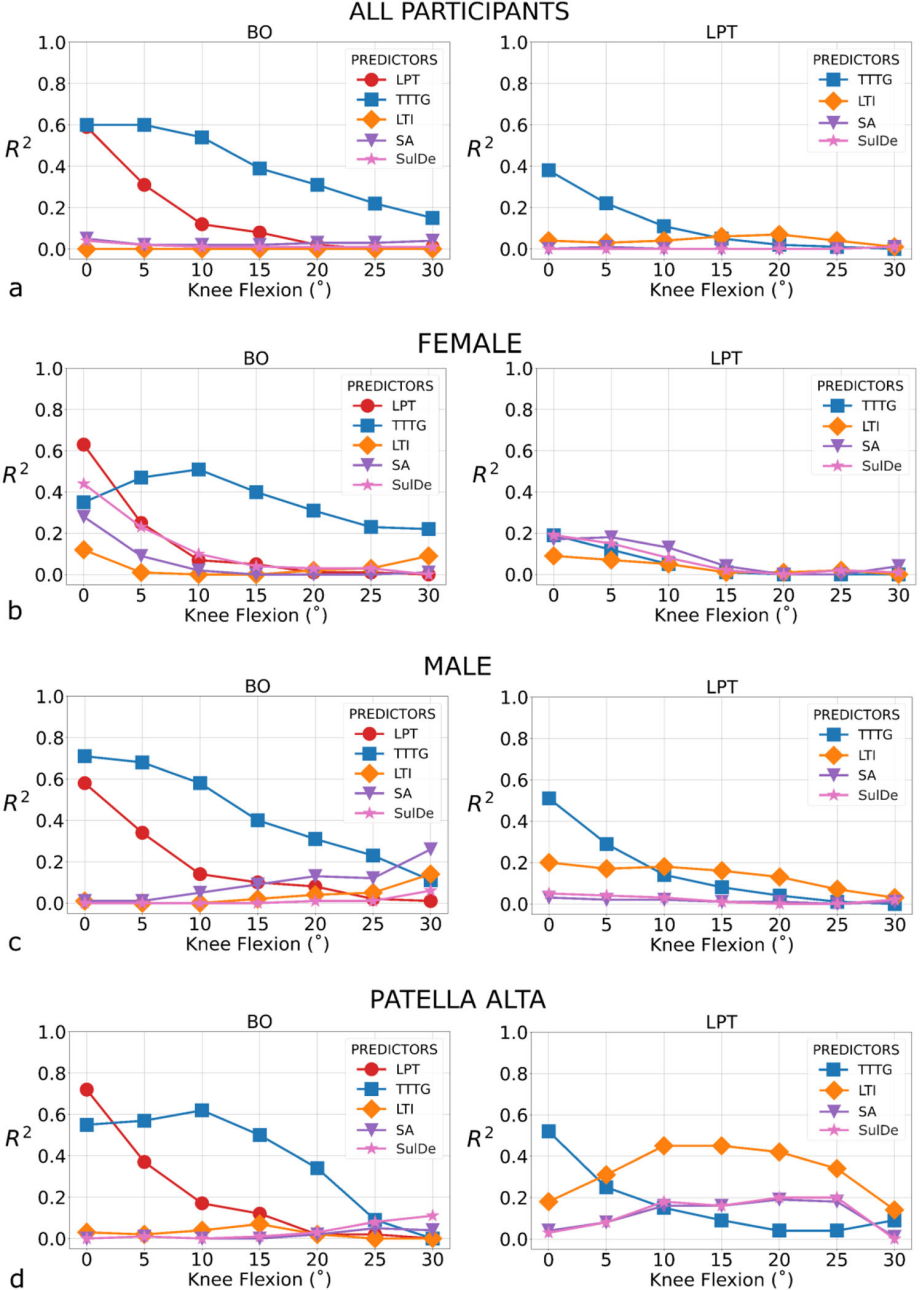
Linear regression (coefficient of determination: *R*^2^) at each flexion angle from 0° to 30° of knee flexion (*x*-axis). **a** All knees together from all participants; stratification by sex for female (**b**) and male (**c**) participants; **d** stratification using only participants that exceed the 1.3 cutoff for patella alta (*n* = 20). TTTG, Tibial tuberosity-tracheal groove; BO, Bisect offset; LPT, Lateral patellar tilt; SA, Sulcus angle; SulDe, Sulcus depth; LTI, Lateral trochlear inclination

Going from full extension to 30° of flexion (eccentric movement), the TTTG decreased gradually by 6.9 mm, the BO by 12.6% and the LPT by 4.3°. During concentric movement between 30° flexion and full extension, the TTTG increased gradually by 6.8 mm, the BO by 14.1% and the LPT by 4.3°. Based on a visual inspection of 95% CI, no significant differences were observed between the eccentric and concentric phases at any flexion angle for all three metrics. Linear regression considering all participants together (Fig. 5a) between kinematics metrics at 0° of knee flexion showed a moderate fit between BO and TTTG (*R*^2^ = 0.60, *p* < 0.001, residual SD = 2.78, β: 1.75, βSE = 0.23) and BO and LPT (*R*^2^ = 0.59, *p* < 0.001, residual SD = 3.27, β = 1.49, βSE = 0.20), and a low fit between TTTG and LPT (*R*^2^ = 0.38, *p* < 0.001, residual SD = 4.04, β = 0.53, βSE = 0.11). Very low non-significant fit was displayed for SA, SulDe, and LTI (*R*^2^ = 0.00–0.05).

When stratified by sex (Fig. 5b, c), male participants showed a strong fit toward 0° of knee flexion between BO and TTTG (*R*^2^ = 0.71, *p* < 0.001, residual SD = 2.66, β = 1.89, βSE = 0.26) while it was low for female participants (*R*^2^ = 0.35, *p* = 0.009, residual SD = 2.80, β = 1.34, βSE = 0.45). In female participants, except for BO and LPT (*R*^2^ = 0.63, *p* < 0.001, residual SD = 3.00, β = 0.50, βSE = 0.10) and BO and SulDe (*R*^2^ = 0.44, *p* = 0.003, residual SD = 5.09, β = −3.05, βSE = 0.87), a very low fit was shown when the relationship between kinematics metrics and morphological metrics was explored. Participants with patella alta (Fig. 5d) displayed a strong fit between BO and LPT (*R*^2^ = 0.72, *p* < 0.001, residual SD = 3.23, β = 1.46, βSE = 0.22) and a moderate fit between BO and TTTG (*R*^2^ = 0.55, *p* < 0.001, residual SD = 2.73, β: 1.91, βSE = 0.41) and LPT and TTTG (*R*^2^ = 0.52, *p* < 0.001, residual SD = 4.23, β = 1.08, βSE =0.25) at 0° of knee flexion. At 10° of flexion, a better fit was shown between BO and TTTG (*R*^2^ = 0.62, *p* < 0.001, residual SD = 2.44, β = 1.62, βSE = 0.32) and between LPT and LTI (*R*^2^: 0.45, *p* = 0.002, residual SD = 3.23, β = 0.44, βSE = 0.12) compared to full extension.

A very low fit was shown for the other morphological metrics (*R*^2^ = 0.00–0.18). A general progressive lower fit was displayed at higher flexion angles with a very low fit across all comparisons at 30° of knee flexion (Fig. 5).

## Discussion

To the best of our knowledge, this is the first study that quantifies three-dimensional knee kinematics of healthy participants during weight-bearing using dynamic CT scanning. This method provides a more realistic assessment of patellar motion under physiological conditions compared to non-weight-bearing techniques. It showed average TTTG, BO, and LPT values of 13.6 mm, 70.1%, and 13.2° at 0° flexion, respectively, and a progressively decreasing trend for all three metrics towards 30° of knee flexion. Such a gradual change was also previously reported using static weight-bearing CT [18], magnetic resonance images [45], and non-weight-bearing four-dimensional CT [27, 28, 30], but with important variability in terms of the magnitude of the parameters and the number of participants investigated.

A very similar pattern was seen between the eccentric and concentric phases. As such, patella tracking metrics could be investigated in either phase within the first 30° of knee flexion. However, pathological knees may demonstrate specific differences between both phases, which needs further investigation.

**Fig. 6:**
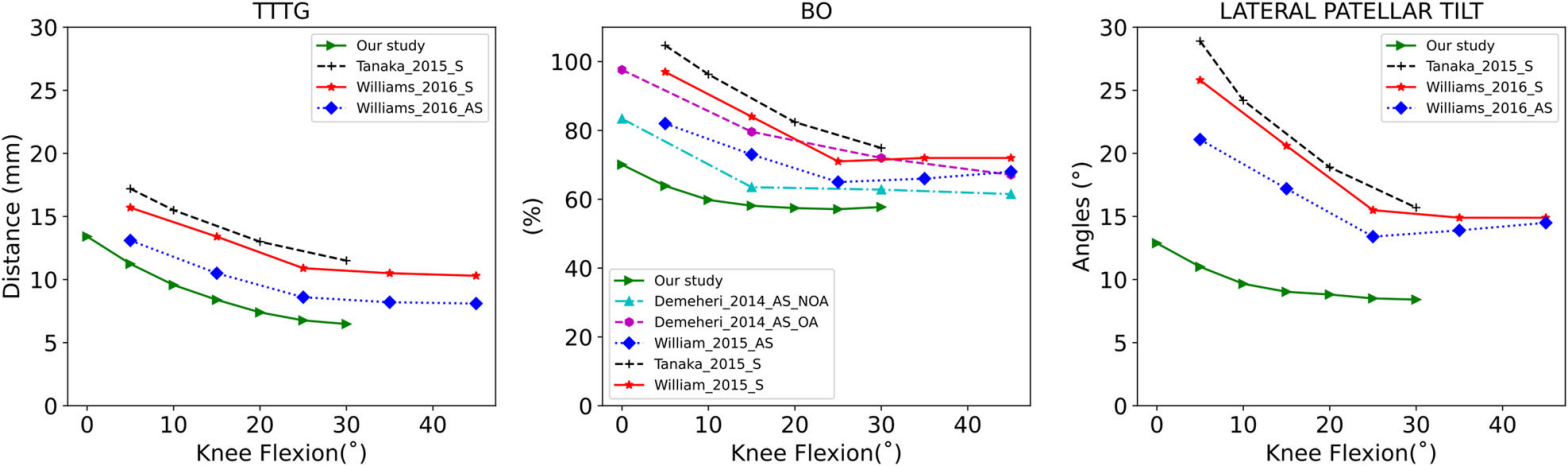
Comparison between healthy subjects of our study and other publications using 4DCT images to investigate patellar tracking. TTTG, Tibial tuberosity-tracheal groove; BO, Bisect offset, LPT, Lateral patellar tilt; S, Symptomatic participants; AS, Asymptomatic participants; AS_OA, Asymptomatic participants with osteoarthrosis (OA); AS_NOA, Asymptomatic participants without osteoarthrosis (NOA)

Our study of healthy participants in weight-bearing conditions reported differences compared to previous dynamic CT studies that investigated both symptomatic and asymptomatic knees without weight-bearing (Fig. 6). Overall, compared to previous studies, we report lower values for all metrics even when looking only at asymptomatic knees. Possible explanations for this difference are the absence of weight-bearing conditions or the fact that we investigated participants with two healthy knees. Indeed, differences between healthy participants and the asymptomatic knee of a patient could reflect altered kinematics on the asymptomatic side. As such, from a kinematic point of view, an asymptomatic knee of a patient might not necessarily be similar to a healthy knee. Overall, TTTG distances, BO, and LPT reported in our study and on other asymptomatic knees [28, 30] were lower compared to those measured on symptomatic knees [27, 28]. However, this was due to larger values in extension and all metrics decrease gradually in a similar way during knee flexion. From 30° of knee flexion and beyond, all metrics reached a plateau whether or not pathology was present. However, the plateau tended to be higher in symptomatic knees but differences between healthy and pathological knees tended to become smaller [27, 28].

**Fig. 7:**
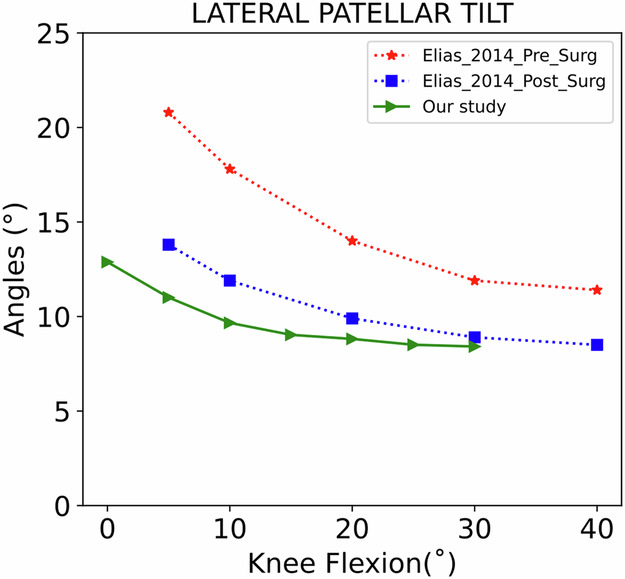
Comparison of the LPT metric between the healthy subjects of our study and pre/post-surgical intervention from Elias et al [47]

Only one study reported changes in LPT before and after surgery using dynamic CT [47]. Compared to our study (Fig. 7), differences between healthy and post-surgical patients are still visible in the first degrees of the knee flexion. However, after 30˚, the two curves seem to converge and reach a plateau. Therefore, in terms of LPT, the surgical intervention seemed able to restore normal patellar kinematics above 30° of knee flexion. However, the study had a small sample size (*n* = 8) and the results were very variable.

Simple linear regression analysis of all participants showed a low to moderate fit between kinematic variables at 0° and very low at 30°. The closer the knee is to full extension, the higher the percentage of the variation in lateral displacement of the patella is explained by lateralisation of the patellar tendon. The lateralisation of the patellar tendon seems to have a substantial impact; in fact, for every millimetre that the patellar tendon was lateralisation, the patella moved 1.75 mm in the lateral direction. The impact on the tilting of the patella was less visible. The overall average values (*R*^2^ = 0.60) were similar to the values reported by Tanaka et al, who reported a moderate fit between TTTG and BO (*R*^2^ = 0.49) and TTTG and LPT (*R*^2^ = 0.52) [27].

When subgrouping by sex, a strong fit was found for male participants between the TTTG and the BO (*R*^2^ = 0.71) and a moderate fit between TTTG and LPT (*R*^2^ = 0.51). This highlighted a greater impact of patellar tendon orientation in male participants, where a one-millimetre increase in the lateralisation of the patellar tendon caused an almost twofold increase in lateral displacement. When only participants with patella alta were considered, a strong fit between BO and LPT (*R*^2^ = 0.72) was reported, and a high impact of the TTTG over BO (β = 1.91) was observed. These findings are in line with those of Conry et al who reported a strong fit between both BO and LPT and between BO and LTI with the knee at full extension (*R*^2^ = 0.84) in participants with patella alta [29]. Based on these findings, the lateralisation of the patellar tendon has a substantial impact on patellar displacement as the knee approaches the last 10° of extension, with a greater impact observed in males or participants with patella alta.

The different morphological metrics describing the depth of the trochlear groove, the flatness of the medial and lateral side and the length of the patella tendon (relative to the size of the patella) showed weak to very weak predictive value to the kinematic metrics with very limited impact on the kinematics metrics. The only exception observed was in the depth of the sulcus in female participants, where a one-millimetre increase in the SulDe causes a threefold reduction in patellar displacement. Overall, it seemed that in healthy participants the morphology of the trochlear groove has minimal impact on the lateral displacement of the patella while performing a weight-bearing squat-like movement. This is in contrast to patients experiencing patellar instability, wherein excessive lateral patellar tracking may predominantly result from either a shallow trochlear groove or a combination of patella alta and a lateral position of the tibial tuberosity [29]. However, all available prediction models only reported the goodness of fit of the model (*R*^2^) and not the impact that one metric has on the other (beta coefficient). Moreover, non-weight-bearing acquisitions might overestimate the impact of this metric due to the limited action of muscle contraction in stabilising patella displacement.

Some limitations need to be addressed. This study only considered the first 30° of knee flexion. It would be interesting to explore the different metrics at higher angles. Due to the limited detector size and diameter of the CT scan gantry, it is difficult to capture more than 40° of knee flexion. Further advancements, such as CT scanners with a larger field of view, are currently being tested and could allow wider movement coverage in the near future. Secondly, we only evaluated 21 participants. As such, our results might not allow defining the full range of normal patella tracking metrics. We believe more subjects, including a more diverse population of healthy subjects, should be evaluated. Finally, to demonstrate the advantage of the weight-bearing protocol, a direct comparison of results with and without the newly presented device should be assessed in the same participants.

In conclusion, weight-bearing dynamic CT of the knee between 0° and 30° is feasible and showed no difference in metrics between eccentric and concentric movements. Lateralisation of the patellar tendon (TTTG) seems to have an important impact on the tracking of the patella, especially in male individuals or those with patella alta. However, except for the depth of the trochlear groove sulcus in females, trochlear groove morphology metrics in healthy participants did not show any added value in predicting patellar lateral displacement and tilt. Further studies will compare these normative values with those from patients to identify abnormal motion patterns and determine whether morphological characteristics significantly impact patella tracking.

## Abbreviations

BO: Bisect offset
CI: Confidence interval
CT: Computed tomography
ISI: Insall–Salvati index
LPT: Lateral patellar tilt
LTI: Lateral trochlear inclination
SA: Sulcus angle
SulDe: Sulcus depth
TTTG: Tibial tuberosity tracheal groove
βSE: Standard error of the β coefficient
